# Patient Safety Culture in a Public Tertiary Care Teaching Hospital in Central India: A Cross-Sectional Study Using the Hospital Survey on Patient Safety Culture (HSOPSC) Version 2.0

**DOI:** 10.7759/cureus.105740

**Published:** 2026-03-23

**Authors:** Nitin Marathe, Abin Varghese, Diksha Patil, Gigini George, Quadri SR, Vishwajeet Singh, Thanga Raj B, Suruchi Rawat

**Affiliations:** 1 Hospital Administration, All India Institute of Medical Sciences, Nagpur, Nagpur, IND; 2 College of Nursing, All India Institute of Medical Sciences, Nagpur, Nagpur, IND; 3 Biostatistics, All India Institute of Medical Sciences, Nagpur, Nagpur, IND; 4 Nursing Services, All India Institute of Medical Sciences, Nagpur, Nagpur, IND

**Keywords:** cross-sectional study, healthcare safety culture, hospital safety climate, hsopsc 2.0, patient safety culture, public tertiary hospital, quality improvement, safety culture assessment, tertiary care hospital, workplace safety perception

## Abstract

Background: Patient safety culture is a critical determinant of healthcare quality and is associated with improved clinical outcomes and patient satisfaction. It can be measured using validated survey instruments assessing organizational norms, behaviors, and healthcare professionals’ perceptions related to safety. In public tertiary care teaching hospitals, where high patient volumes, workforce constraints, and training responsibilities coexist, evaluating patient safety culture is particularly important. Standardized tools such as the Hospital Survey on Patient Safety Culture (HSOPSC) Version 2.0 enable identification of strengths, gaps, and context-specific priorities for improvement.

Objective: To assess patient safety culture in a public tertiary care teaching hospital in Central India using HSOPSC Version 2.0 and identify strengths and gaps across key safety culture dimensions at unit and hospital levels, including variations across professional groups and clinical departments.

Methods: A descriptive cross-sectional study was conducted in accordance with Strengthening the Reporting of Observational Studies in Epidemiology guidelines at a public tertiary care teaching hospital in Central India. Patient safety culture was assessed using HSOPSC Version 2.0 through a mixed-mode (online and paper-based) survey. Doctors, nurses, and technical staff with at least six months of institutional experience were included. Proportionate stratified sampling yielded 414 respondents (response rate: 80%). Data were analyzed using the Agency for Healthcare Research and Quality (AHRQ) HSOPSC 2.0 Data Entry and Analysis Tool. Percent-positive and composite dimension scores were calculated according to AHRQ guidance. Internal consistency was assessed using Cronbach's alpha. One-way analysis of variance was used for comparisons, with p < 0.05 considered statistically significant.

Results: A total of 414 healthcare professionals participated (response rate: 80%). Overall, the patient safety culture was moderate. Teamwork (77.54%) and communication about error (76.31%) were identified as strengths, followed by organizational learning-continuous improvement (69.01%), supervisor/clinical leader support (68.58%), and handoffs and information exchange (60.11%), which demonstrated moderate performance. Staffing and work pace (32.25%) was identified as an area of concern, indicating an important system-level challenge, while response to error (50.00%) was at the threshold of the moderate range. Physicians reported significantly more positive perceptions than nurses and residents across several domains (p < 0.05). Significant variation in safety culture scores was also observed across work areas/units. Overall, 84.30% of respondents (n=349) rated patient safety in their work area as good to excellent.

Conclusion: This study provides structured evidence on patient safety culture in a public tertiary care teaching hospital in Central India. While strengths in teamwork, communication about error, organizational learning, and clinical leadership support provide a foundation for safe care, persistent system-level challenges, including staffing constraints, workload pressures, care transition gaps, and concerns regarding non-punitive response to error, require focused leadership attention. Targeted, context-specific interventions and sustained organizational commitment are essential to advance safety culture maturity in resource-constrained public healthcare settings.

## Introduction

Unsafe healthcare remains a major global public health challenge. The landmark report To Err Is Human first quantified the scale of preventable adverse events in modern healthcare systems, catalyzing the global patient safety movement [1]. Subsequent international evidence has demonstrated that patient harm occurs across health systems, with a substantial proportion considered preventable [2,3]. The World Health Organization (WHO) has further emphasized patient safety as central to health system strengthening, including through the Global Patient Safety Action Plan 2021-2030 [4]. Beyond individual adverse events, organizational culture exerts an important influence on safety outcomes. Positive safety culture has been associated with improved clinical processes, stronger reporting behaviors, reduced adverse events, and greater patient satisfaction [5,6].

Patient safety culture refers to the shared values, attitudes, perceptions, and patterns of behavior that determine an organization's commitment to safety. Recognizing that safety outcomes are largely shaped by system-level factors rather than individual failings, healthcare organizations have adopted standardized tools to measure safety culture and guide improvement [6]. Among these, the Hospital Survey on Patient Safety Culture (HSOPSC), developed by the Agency for Healthcare Research and Quality (AHRQ), is one of the most widely used instruments internationally [7]. The HSOPSC has undergone extensive psychometric evaluation and has been translated, adapted, and validated across multiple countries, demonstrating acceptable reliability and construct validity in diverse settings [7,8]. Its revised Version 2.0 was developed to improve measurement clarity, streamline composite dimensions, and strengthen its utility for benchmarking and quality improvement initiatives [9].

Cross-national validation studies have confirmed acceptable psychometric properties of the HSOPSC across diverse healthcare settings. A large-scale psychometric evaluation conducted across Swedish hospitals and primary care settings (n > 84,000) demonstrated variability in internal consistency across dimensions, with staffing-related composites consistently yielding lower reliability coefficients, a pattern observed across multiple cultural adaptations [10]. A comprehensive systematic review of HSOPSC studies further established that teamwork within units is the most consistently high-scoring dimension globally, while non-punitive response to error and staffing adequacy represent the most persistently low-scoring dimensions across health systems [11].

Despite expanding attention to safety culture globally, healthcare systems in low- and middle-income countries (LMICs) face distinct structural and operational challenges. Resource limitations, workforce shortages, high patient volumes, and hierarchical organizational structures may influence reporting behaviors and perceptions of safety [12]. Studies from LMIC tertiary hospitals have frequently reported lower scores in domains such as staffing adequacy and non-punitive response to error, reflecting systemic pressures and cultural barriers to transparent reporting [13,14]. These findings underscore the importance of context-sensitive assessments that accurately reflect organizational realities in resource-constrained environments.

A systematic review and meta-analysis of HSOPSC studies from Latin American hospitals (30 studies, n = 10,915) confirmed that teamwork within units and organizational learning were the strongest dimensions across five countries, while non-punitive response to error and staffing consistently ranked among the weakest--underscoring shared structural determinants of safety culture in resource-constrained environments [15]. A large cross-sectional HSOPSC study conducted across 32 hospitals in China (n > 1,160 participants) similarly reported persistently low staffing-related scores, with meaningful variation across professional roles and hospital types--patterns broadly consistent with findings from other LMICs [16].

In India, public tertiary care teaching hospitals provide multispecialty services while also supporting undergraduate and postgraduate training in high-volume care environments [17]. Qualitative work from government facilities has described how resource constraints (including inadequate staffing and overcrowding) can coexist with professional cultures marked by punitive responses to adverse events and rigid workplace hierarchies--contextual features that may shape psychological safety, communication openness, and reporting/accountability behaviors [18]. National policy initiatives, such as the Ministry of Health and Family Welfare’s National Patient Safety Implementation Framework, have increased the visibility of patient safety within public services; however, operational evaluations suggest that structural support systems and patient safety practices vary across public facilities [19]. Despite growing policy and quality-improvement attention, published institution-level assessments of patient safety culture in Indian public-sector tertiary hospitals remain limited, with HSOPSC-based surveys reported from a relatively small number of public tertiary centers [17].

Professional role has been identified as a significant determinant of safety culture perceptions in HSOPSC studies conducted in hierarchically organized healthcare settings. A cross-sectional study among registered nurses in Omani public hospitals (n = 414) using the HSOPSC demonstrated that nurses' overall patient safety perceptions were significantly associated with perceived supervisor expectations, feedback and communication about errors, and teamwork across hospital units [20]. These findings reinforce the importance of examining interprofessional variation in safety culture assessments conducted in complex teaching environments such as Indian public-sector tertiary hospitals, where hierarchical role structures may meaningfully shape safety perceptions across groups.

Furthermore, while earlier studies in India have utilized previous HSOPSC versions, evidence using HSOPSC Version 2.0 in Indian public tertiary hospitals remains sparse. There is limited published work examining variation in safety culture across professional groups and clinical departments within Indian public institutions. Such analysis is important because safety perceptions may differ across hierarchical roles and functional units, directly influencing the design and impact of improvement strategies. Therefore, the primary objective of this study was to assess patient safety culture in a public tertiary care teaching hospital in Central India using HSOPSC Version 2.0. Secondary objectives included identifying strengths and areas for improvement and examining variations across professional groups and work areas/units.

## Materials and methods

Study design and reporting standards

A descriptive cross-sectional study was conducted at a public tertiary care teaching hospital in Central India between March and July 2025. A cross-sectional survey design was employed, as it allows systematic assessment of healthcare professionals’ perceptions of patient safety culture at a single point in time using validated instruments such as HSOPSC, which are designed for such applications. The study was designed and reported in accordance with the Strengthening the Reporting of Observational Studies in Epidemiology (STROBE) [21] guidelines for cross-sectional studies. A mixed-mode survey strategy incorporating both online and paper-based formats was employed to optimize participation across professional groups. The mode of response was determined by participant preference.

Study setting

The study was carried out at the All India Institute of Medical Sciences (AIIMS), Nagpur, a public tertiary care referral hospital serving a large population in Central India. The institution functions as both a clinical service provider and a teaching hospital. At the time of the study, the hospital recorded an average daily outpatient attendance of approximately 4,000 patients and comprised 16 inpatient wards representing a broad range of medical and surgical specialties.

Study population and eligibility criteria

The study population consisted of frontline healthcare professionals directly involved in patient care and patient safety activities. Eligible participants included doctors, nurses, and technical healthcare staff, including pharmacists, laboratory technicians, dialysis technicians, and operating theater technicians. Participants were required to have at least six months of institutional experience to ensure adequate familiarity with hospital processes and prevailing safety practices. Healthcare students and trainees were excluded to ensure that responses reflected perceptions grounded in sustained professional engagement within the organization.

Sampling strategy and sample size

A comprehensive sampling frame was developed using institutional administrative records. Proportionate stratified sampling was employed to ensure adequate representation across professional categories. A total of 520 eligible healthcare professionals were invited to participate. Of these, 414 completed the survey, yielding a response rate of 80%.

The sample size was calculated using OpenEpi (Version 3.01; www.OpenEpi.com) [22]. The calculation was based on an estimated proportion of positive patient safety culture of 58%, with a 95% confidence level and an absolute precision of 5%. The estimated proportion of 58% was derived from previously published HSOPSC-based studies conducted in similar tertiary care settings. To account for potential non-response, an additional 10% was added, resulting in a minimum required sample size of 413 participants.

Data collection instrument

Patient safety culture was assessed using the Hospital Survey on Patient Safety Culture (HSOPSC) Version 2.0, developed by the Agency for Healthcare Research and Quality (AHRQ) [9]. The HSOPSC 2.0 consists of 34 items that assess patient safety culture across 12 dimensions, encompassing 10 safety culture composites and two outcome measures. Items are scored on a five-point Likert agreement scale ("strongly disagree" to "strongly agree") or a five-point frequency scale ("never" to "always"). Both positively and negatively worded items are included. The two outcome measures assessed the overall patient safety grade in the respondent's work area and the frequency of event reporting during the preceding 12 months. Demographic and work-related variables collected included professional role, clinical unit, tenure at the hospital and current unit, weekly working hours, and direct patient contact. The questionnaire was administered in English, which is the standard medium of professional communication in the study setting, and all participants were proficient in English.

Data collection procedure

Data collection was conducted over a five-month period from March to July 2025. Prior to survey administration, informational material was disseminated across clinical units to increase awareness and encourage participation. The online survey was initially distributed via institutional email. Reminder notifications were issued during the second and third weeks of the data collection period, followed by in-person follow-up where required. If the response rate remained below 50% after four weeks, an additional reminder was circulated, and the data collection period was extended. The mixed-mode approach was adopted to improve response coverage across professional groups and clinical settings.

Ethical considerations

Ethical approval was obtained from the Institutional Ethics Committee of AIIMS Nagpur (IEC reference: IEC/Pharmac/2025/1143). Participation was entirely voluntary, and completion of the questionnaire was considered implied informed consent. The survey was administered anonymously to reduce social desirability bias. No identifying information was collected from participants.

Data analysis

Data were entered and analyzed using the AHRQ HSOPSC Version 2.0 Data Entry and Analysis Tool for Microsoft Excel (Microsoft Corporation, Redmond, Washington, USA). Descriptive statistics were used to summarize participant characteristics, individual item responses, composite dimension scores, and outcome measures. Percent-positive scores were calculated in accordance with AHRQ guidelines. For positively worded items, percent-positive responses represented the proportion of respondents selecting "strongly agree" or "agree," or "most of the time" or "always" for frequency-based items. For negatively worded items, percent-positive responses represented the proportion selecting "strongly disagree" or "disagree." Composite scores for each safety culture dimension were calculated by averaging percent-positive responses across items within the respective dimension, excluding missing values. Internal consistency reliability was assessed using Cronbach's alpha. Comparisons across professional groups and clinical departments were conducted using one-way analysis of variance (ANOVA). Statistical significance was set at p < 0.05.

## Results

Participant characteristics

A total of 414 healthcare professionals participated in the study (response rate: 80%). Registered nurses constituted the largest professional group (n = 203, 49.03%), followed by residents (n = 91, 21.98%), physicians (n = 64, 15.46%), and others (n = 56, 13.53%). Among the 410 participants who reported their work areas/units, 117 (28.54%) worked in medical (non-surgical) units, 89 (21.71%) in surgical units, 95 (23.17%) in combined/specialty units, and 109 (26.59%) in other units. Regarding tenure at the hospital, 112 (27.05%) had worked for <1 year, 292 (70.53%) for one to five years, and 10 (2.42%) for ≥6 years. For tenure in the current unit, 155 (37.44%) had worked for <1 year, 249 (60.14%) for one to five years, and 10 (2.42%) for ≥6 years. Direct patient contact was reported by 365 (88.59%) of the 412 respondents, while 47 (11.41%) reported no direct patient contact. Regarding events reported in the past year, 215 (51.93%) reported no events, 128 (30.92%) reported one to two events, and 71 (17.15%) reported more than two events. Detailed participant characteristics are presented in Table 1.

**Table 1:** Participant characteristics (N = 414). *Work areas/units: Data available for 410 respondents. Units were grouped into medical (non-surgical), surgical (including peri-operative), combined/specialty (combined medical-surgical, obstetrics, pediatrics, ICU), and other units (diagnostic, administrative, and support services) for analysis. †Direct patient contact: Data available for 412 respondents.

Variable	Category	n (%)
Professional role	Registered nurses	203 (49.03%)
	Residents	91 (21.98%)
	Physicians	64 (15.46%)
	Others	56 (13.53%)
	Total	414 (100%)
Work areas/units*	Medical units (non-surgical)	117 (28.54%)
	Surgical units	89 (21.71%)
	Combined/specialty units	95 (23.17%)
	Other units	109 (26.59%)
	Total	410 (100%)
Tenure at hospital	<1 year	112 (27.05%)
	1–5 years	292 (70.53%)
	≥6 years	10 (2.42%)
	Total	414 (100%)
Tenure in current unit	<1 year	155 (37.44%)
	1–5 years	249 (60.14%)
	≥6 years	10 (2.42%)
	Total	414 (100%)
Direct patient contact†	Yes	365 (88.59%)
	No	47 (11.41%)
	Total	412 (100%)
Events reported in past year	0	215 (51.93%)
	1–2	128 (30.92%)
	>2	71 (17.15%)
	Total	414 (100%)

Composite patient safety culture scores

Composite percent-positive scores across the ten HSOPSC 2.0 dimensions are presented in Table 2. Teamwork demonstrated the highest composite percent-positive score (77.54%), followed by communication about error (76.31%), organizational learning-continuous improvement (69.01%), and supervisor/clinical leader support (68.58%). Consistent with commonly used HSOPSC interpretive conventions, scores of ≥75% may be considered areas of strength, scores between 50% and 75% indicate moderate performance, and scores of <50% suggest areas of concern; these thresholds are interpretive rather than definitive. Accordingly, teamwork and communication about errors were identified as strengths. Organizational learning-continuous improvement, supervisor/clinical leader support, communication openness (63.12%), reporting patient safety events (61.59%), handoffs and information exchange (60.11%), and hospital management support (54.45%) fell within the moderate range. Response to error (50.00%) was at the threshold of the moderate range, indicating a potential area requiring attention, whereas staffing and work pace (32.25%) represented the most prominent area of concern. Detailed item-level response distributions are provided in the Appendix.

**Table 2:** Composite dimension scores and internal consistency reliability (N = 414).

Dimension	Mean (SD)	% Positive (SD)	Cronbach's alpha
Teamwork	3.96 (0.78)	77.54 (29.27)	0.541
Communication about error	4.07 (0.88)	76.31 (34.94)	0.773
Organizational learning—continuous improvement	3.69 (0.84)	69.01 (34.50)	0.609
Supervisor/clinical leader support	3.65 (0.78)	68.58 (31.07)	0.604
Communication openness	3.73 (0.95)	63.12 (35.97)	0.753
Reporting patient safety events	3.57 (1.12)	61.59 (43.08)	0.719
Handoffs and information exchange	3.48 (0.83)	60.11 (38.52)	0.547
Hospital management support	3.32 (0.83)	54.45 (34.55)	0.527
Response to error	3.21 (0.86)	50.00 (34.43)	0.674
Staffing and work pace	2.70 (0.73)	32.25 (25.58)	0.386

Internal consistency

Cronbach's alpha values ranged from 0.386 to 0.773 (Table 2). Acceptable internal consistency (α ≥ 0.70) was observed for communication about error (α = 0.773), communication openness (α = 0.753), and reporting patient safety events (α = 0.719). Lower reliability coefficients were observed for staffing and work pace (α = 0.386), hospital management support (α = 0.527), handoffs and information exchange (α = 0.547), and teamwork (α = 0.541).

Differences in patient safety culture scores by professional category

Differences in patient safety culture composite scores across professional categories are presented in Table 3. Statistically significant differences were observed across all ten dimensions (p < 0.05). Registered nurses reported the highest mean scores for teamwork (4.06 ± 0.67) and handoffs and information exchange (3.63 ± 0.83). Physicians reported the highest scores for communication about error (4.27 ± 0.73), communication openness (4.02 ± 0.85), organizational learning (3.88 ± 0.65), supervisor/clinical leader support (4.13 ± 0.67), and reporting patient safety events (4.10 ± 0.86). Residents reported the highest scores for response to error (3.46 ± 0.81) and staffing and work pace (3.03 ± 0.63), whereas other staff reported the highest score for hospital management support (3.70 ± 0.72).

**Table 3:** Differences in patient safety culture scores by professional category (mean ± SD). Values reported as mean (SD). One-way ANOVA; p < 0.05 is considered statistically significant.

Dimension	Registered nurse (n = 203)	Resident (n = 91)	Physician (n = 64)	Others (n = 56)	p-value
Teamwork	4.06 (0.67)	3.91 (0.75)	3.96 (0.89)	3.70 (0.97)	0.022
Communication about error	4.19 (0.89)	3.85 (0.87)	4.27 (0.73)	3.76 (0.86)	<0.001
Communication openness	3.74 (1.04)	3.61 (0.81)	4.02 (0.85)	3.58 (0.88)	0.036
Organizational learning	3.56 (0.89)	3.83 (0.68)	3.88 (0.65)	3.71 (0.96)	0.012
Supervisor/clinical leader support	3.47 (0.81)	3.74 (0.68)	4.13 (0.67)	3.60 (0.69)	<0.001
Hospital management support	3.11 (0.88)	3.40 (0.76)	3.58 (0.66)	3.70 (0.72)	<0.001
Reporting patient safety events	3.45 (1.23)	3.42 (1.00)	4.10 (0.86)	3.60 (0.99)	<0.001
Response to error	3.05 (0.90)	3.46 (0.81)	3.39 (0.77)	3.21 (0.77)	<0.001
Staffing and work pace	2.54 (0.72)	3.03 (0.63)	2.71 (0.63)	2.70 (0.83)	<0.001
Handoffs & Information Exchange	3.63 (0.83)	3.30 (0.81)	3.37 (0.85)	3.34 (0.76)	0.006

Differences in patient safety culture scores by work areas/units

Differences in patient safety culture composite scores across work areas/units are shown in Table 4. Significant differences were observed for most dimensions (p < 0.05), except for teamwork (p = 0.068) and communication about error (p = 0.096). Combined/specialty units reported the highest scores for communication openness (3.91 ± 0.79) and handoffs and information exchange (3.62 ± 0.74). Other units reported the highest scores for organizational learning (3.92 ± 0.61), supervisor/clinical leader support (3.81 ± 0.63), hospital management support (3.56 ± 0.74), response to error (3.35 ± 0.78), and staffing and work pace (2.96 ± 0.71). Surgical units reported the highest score for reporting patient safety events (3.76 ± 0.96).

**Table 4:** Differences in patient safety culture scores by work area/unit (mean ± SD). Values reported as mean (SD). One-way ANOVA; p < 0.05 is considered statistically significant.

Dimension	Medical units (n = 117)	Surgical units (n = 89)	Combined/speciality units (n = 95)	Others (n = 109)	p-value
Teamwork	4.10 (0.68)	3.87 (0.91)	4.01 (0.75)	3.86 (0.77)	0.068
Communication about error	3.95 (0.96)	4.10 (0.94)	4.24 (0.78)	4.01 (0.80)	0.096
Communication openness	3.43 (1.18)	3.85 (0.79)	3.91 (0.79)	3.79 (0.88)	<0.001
Organizational learning	3.36 (1.01)	3.63 (0.88)	3.86 (0.63)	3.92 (0.61)	<0.001
Supervisor/clinical leader support	3.48 (1.01)	3.65 (0.65)	3.65 (0.69)	3.81 (0.63)	0.016
Hospital management support	2.90 (1.02)	3.48 (0.64)	3.42 (0.63)	3.56 (0.74)	<0.001
Reporting patient safety events	3.30 (1.25)	3.76 (0.96)	3.65 (1.11)	3.59 (1.07)	0.026
Response to error	2.89 (0.98)	3.31 (0.78)	3.34 (0.77)	3.35 (0.78)	<0.001
Staffing and work pace	2.46 (0.73)	2.55 (0.69)	2.83 (0.68)	2.96 (0.71)	<0.001
Handoffs and information exchange	3.32 (0.93)	3.60 (0.85)	3.62 (0.74)	3.42 (0.74)	0.026

Overall patient safety rating

**Figure 1:**
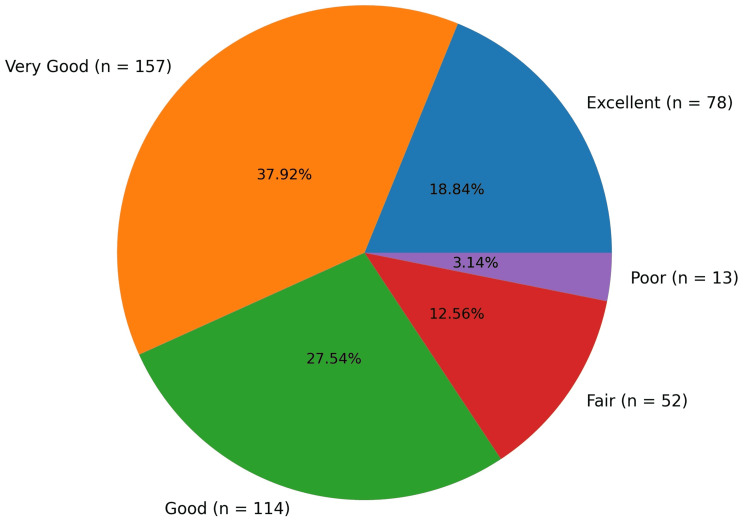
Distribution of overall patient safety ratings (N = 414). Values represent the number of respondents (n) and the corresponding percentage for each category.

Overall patient safety ratings were predominantly positive (Figure 1): 18.84% rated patient safety as excellent (n = 78), 37.92% as very good (n = 157), 27.54% as good (n = 114), 12.56% as fair (n = 52), and 3.14% as poor (n = 13). In total, 84.30% of respondents (n = 349) rated patient safety in their work area as good, very good, or excellent.

## Discussion

Summary of key findings

This study assessed patient safety culture in a public tertiary care teaching hospital using HSOPSC Version 2.0 and identified a moderate overall safety culture profile. Stronger dimensions were observed in teamwork and communication-related constructs, whereas comparatively weaker dimensions included staffing and work pace, response to error, and handoffs and information exchange. Significant differences in safety perceptions were identified across professional groups and clinical departments, and internal consistency varied across dimensions. These findings reflect a safety culture pattern characterized by relatively functional intra-unit collaboration alongside persistent structural and organizational challenges.

Interpretation of strong dimensions

Teamwork emerged as the strongest dimension. HSOPSC studies consistently report relatively higher scores for within-unit teamwork compared to system-level dimensions, although specific patterns vary by setting and instrument version [8,23]. These findings may reflect effective interpersonal collaboration, mutual support during high-acuity periods, and adaptive problem-solving at the unit-level features that are particularly important in complex, high-volume tertiary environments.

International benchmarking evidence corroborates this finding. A systematic review of HSOPSC studies across international settings confirmed that teamwork within units is the most consistently highest-scoring dimension globally, a pattern that holds across diverse national contexts, instrument versions, and hospital types [11]. In the Latin American HSOPSC meta-analysis (30 studies, five countries, n = 10,915), teamwork within units likewise emerged as one of the strongest dimensions across all included settings, suggesting that intra-unit cohesion is a relatively universal feature of hospital safety culture profiles [15].

Communication about error and communication openness also demonstrated comparatively strong performance. These dimensions are widely regarded as important enablers of organizational learning, as they support identification and discussion of risks and improvement opportunities [24]. However, it should be noted that perceived openness in communication does not necessarily equate to a fully non-punitive or psychologically safe reporting environment, as demonstrated by the comparatively lower scores in the response-to-error dimension.

The relatively favorable perception of supervisor or clinical leader support suggests visible frontline leadership engagement, which has been associated with improvements in safety culture when embedded within structured quality improvement efforts [24]. Evidence regarding specific leadership mechanisms, however, remains heterogeneous across settings.

Interpretation of weak dimensions

Staffing and work pace demonstrated the lowest performance in this study (32.25%). This dimension consistently scores lowest across HSOPSC studies in diverse international and LMIC settings [23]. In public-sector tertiary hospitals, high patient volumes and workforce constraints are structural realities that exert ongoing pressure on staff. Observational evidence has demonstrated associations between nurse staffing levels and patient mortality, burnout, and job dissatisfaction [25], underscoring that workload pressures represent safety-relevant system characteristics rather than solely administrative concerns. These observed associations, however, do not establish causality, and multiple confounding factors should be considered.

This finding is further contextualized by systematic evidence from LMIC settings. In the Latin American HSOPSC meta-analysis (30 studies, five countries), staffing consistently emerged as one of the weakest dimensions across all included settings, reflecting structural workforce challenges common to healthcare systems with resource constraints [15]. Similarly, a multi-hospital HSOPSC study conducted across Chinese tertiary and secondary hospitals identified staffing as one of the most persistently low-scoring dimensions, confirming that staffing adequacy is a shared safety culture challenge in resource-limited healthcare environments internationally [16].

The response-to-error dimension also demonstrated comparatively lower positivity (50.00%). Non-punitive response to error has consistently emerged as one of the lowest-scoring dimensions across HSOPSC studies internationally, including in LMIC hospital settings [13,23]. Lower perceptions in this dimension may reflect concerns about blame, perceived personal consequences of reporting, or variability in incident review feedback mechanisms. Such findings should not be interpreted as an absence of reporting systems; rather, they signal potential opportunities to strengthen just culture principles, reinforce psychological safety, and improve the feedback loop following safety event reporting.

Handoffs and information exchange also demonstrated weaker performance (60.11%). Transitions of care are recognized as high-risk points within hospital systems where communication gaps may contribute to adverse events. Implementation of structured handoff protocols has been associated with significant reductions in medical errors and preventable adverse events in academic medical centers [26]. In complex tertiary teaching hospitals with rotating teams and multidisciplinary care pathways, standardized transition communication tools may be particularly relevant to mitigate information loss during handoffs.

Professional and departmental variation

Differences observed across professional categories are consistent with prior HSOPSC research demonstrating variability in safety culture perceptions by role [8,23]. Physicians reported more positive perceptions than nurses and residents across multiple dimensions, a pattern that may reflect differences in workload exposure, hierarchical position, decision-making authority, and daily interaction with formal safety processes. Nurses, who constitute the largest professional group and are most directly exposed to operational pressures, reported comparatively lower scores--findings with direct implications for targeted workforce-specific interventions.

Cross-cultural evidence further supports this nurse-physician divergence. In an HSOPSC study among registered nurses in Omani public hospitals (n = 414), nurses' overall safety culture perceptions were significantly associated with perceived supervisor expectations, feedback about errors, and teamwork across hospital units--highlighting that nursing-specific safety culture perceptions are particularly sensitive to operational environment factors and supervisor-mediated pathways [20]. This evidence reinforces the direct applicability of targeted, role-specific safety culture interventions in hierarchically organized clinical environments such as Indian public teaching hospitals.

Department-level variation further highlights that safety culture is not monolithic within institutions [8]. Local workflow complexity, case mix, staffing patterns, and supervisory practices may shape unit-level safety perceptions. Notably, medical units demonstrated the lowest staffing and work pace scores and the weakest response-to-error perceptions, potentially reflecting the distinctive pressures of high-volume medical wards. Variation was also observed in organizational learning and handoffs across departments, whereas teamwork perceptions were relatively consistent across units. These findings support department-specific feedback and tailored improvement strategies rather than reliance solely on aggregated institutional scores.

Internal consistency

Internal consistency varied across dimensions, with three composites achieving acceptable reliability (α ≥ 0.70) and several below conventional thresholds. Prior psychometric evaluations of HSOPSC have reported similar variability, particularly in staffing and work pace domains [7]. These composites encompass heterogeneous operational elements-such as staffing adequacy, workload intensity, and reliance on temporary personnel-which may reduce coefficient alpha despite clear conceptual relevance. Large-scale validation studies, including a Swedish national evaluation by Hedsköld et al., have documented comparable patterns, attributing lower alpha values in staffing-related domains to multidimensional item structure rather than construct invalidity [10]. Accordingly, the observed variability in reliability should be interpreted in light of established instrument characteristics.

Comparison with international Hospital Survey on Patient Safety Culture (HSOPSC) literature

The overall profile aligns with published HSOPSC literature, which reports relatively stronger teamwork dimensions and persistent challenges in staffing adequacy and in a non-punitive response to error [23]. Similar findings have been reported in Indian tertiary care contexts as well as hospital settings across other LMICs [13,17]. Resource limitations and organizational hierarchy are frequently discussed as potential contributors to variation in safety perceptions; however, cross-sectional survey data do not establish causal pathways and should be interpreted accordingly. Systematic review evidence suggests that safety culture improvements are more reliably achieved when embedded within leadership engagement and structured quality improvement initiatives rather than relying on isolated educational interventions [24].

Systematic review evidence strengthens the international benchmarking context of the current findings. Reis et al. conducted a systematic review of HSOPSC studies internationally and confirmed that positive safety culture profiles characterized by strong teamwork and persistent weaknesses in staffing adequacy and non-punitive response to error represent the most commonly reported pattern globally, across diverse country income levels and hospital types [11]. The Latin American HSOPSC meta-analysis by Camacho-Rodríguez et al. similarly documented these cross-national patterns across 30 studies, further establishing that public hospital safety culture profiles in LMICs share core structural vulnerabilities that are amenable to system-level intervention [15].

Implications for practice

The observed safety culture profile suggests several system-level considerations for public tertiary teaching hospitals in India. First, with respect to workforce planning, staffing-related findings from this survey may inform workload assessment, duty-hour structures, and human resource allocation strategies at both departmental and institutional levels. Second, strengthening a non-punitive reporting culture may require consistent leadership communication emphasizing learning from error, transparent event review processes, and clear differentiation between learning-oriented review mechanisms and disciplinary procedures. Third, implementation of structured communication tools and protected transition time during handoffs may help reduce perceived information gaps at care transitions. Fourth, formalizing structured feedback loops that build on existing communication strengths may enhance institutional learning and reinforce safety culture foundations. Given the dual service and teaching mandate of public tertiary hospitals, safety culture interventions should be designed to accommodate clinical complexity, training hierarchies, and prevailing workforce constraints.

Evidence on effective strategies for improving patient safety culture provides additional direction for these system-level recommendations. A systematic review by Morello et al. examining interventions to improve safety culture in hospital settings found that multicomponent programs-combining leadership engagement, staff training, structured feedback mechanisms, and quality improvement frameworks-were more consistently associated with improvements in safety culture compared with single-component interventions [27]. Discrete or isolated educational initiatives demonstrated limited and inconsistent effects. These findings suggest that coordinated, sustained organizational strategies may be more appropriate when addressing the multidimensional safety culture gaps identified in the present study.

Strengths

This study utilized HSOPSC Version 2.0, enabling contemporary benchmarking with emerging international literature using the updated instrument. Inclusion of multiple professional categories, such as nurses, physicians, residents, and technical staff, enhances the practical applicability of findings. Department-level analysis provides granular, actionable data for institutional quality improvement. Use of standardized AHRQ scoring methodology improves comparability and methodological transparency. The high response rate (80%) strengthens the representativeness of findings.

Limitations

Several limitations should be considered when interpreting these findings. First, the cross-sectional design precludes causal inference and limits the ability to establish relationships between variables, as the results reflect self-reported perceptions captured at a single point in time. Second, responses may have been influenced by social desirability bias; however, anonymous survey administration and mixed-mode delivery were employed to reduce this risk. Third, the single-center design limits generalizability to institutions with differing governance structures, resource availability, and organizational cultures. Fourth, variability in internal consistency across certain dimensions-particularly staffing and work pace (α = 0.386)-should be considered when interpreting those composite scores. Fifth, although the study included multiple professional groups, the distribution of respondents was uneven, with nurses representing the largest proportion, which may influence the statistical power of interprofessional comparisons. Finally, no post-hoc correction for multiple comparisons was applied in the exploratory departmental and professional group analyses; therefore, these results should be interpreted with appropriate caution.

Future directions

Longitudinal assessment would enable evaluation of safety culture trends over time and the impact of targeted interventions. Qualitative exploration of perceptions surrounding response to error and staffing pressures may clarify the contextual factors that shape survey responses. Formal validation of the HSOPSC 2.0 instrument within the Indian public hospital context would strengthen its psychometric basis for this population. Multi-centre studies across Indian public-sector hospitals would enhance generalizability and enable system-level benchmarking.

## Conclusions

This study provides structured evidence on patient safety culture in a public tertiary care teaching hospital in Central India using HSOPSC Version 2.0. The safety culture profile demonstrated strengths in intra-unit teamwork and communication alongside structural challenges related to staffing adequacy, response to error, and care transitions. Addressing these multidimensional gaps may require coordinated system-level action encompassing workforce planning, leadership engagement, structured organizational learning mechanisms, and standardized handoff processes. Advancing safety culture maturity in similar public tertiary teaching hospitals will likely depend on sustained organizational commitment to these priorities. Strengthening these elements may enhance staff confidence in reporting systems and institutional safety resilience within resource-constrained public healthcare settings. These findings provide a contextually relevant foundation for institutional safety improvement initiatives and may inform future multi-center benchmarking efforts across Indian public-sector tertiary hospitals.

## Appendices

**Table 5:** Supplementary table: Item-level response distribution for the Hospital Survey on Patient Safety Culture (HSOPSC) Version 2.0 (N = 414). Items marked with an asterisk (*) are negatively worded and were reverse-coded during calculation of composite percent-positive scores in accordance with Agency for Healthcare Research and Quality (AHRQ) HSOPSC Version 2.0 scoring guidelines. Note: All values represent item-level response distributions from the current study sample (N = 414) only and do not incorporate external benchmark data from AHRQ or other institutions. Item-level totals may vary slightly across items because missing responses were excluded from percent-positive calculations in accordance with AHRQ HSOPSC Version 2.0 scoring guidance. Values represent valid responses for each item.

Dimension	Item (brief description)	Positive n (%)	Neutral n (%)	Negative n (%)
Teamwork	A1 Staff work together as an effective team	365 (88.38)	25 (6.05)	23 (5.57)
	A8 Staff help each other during busy times	326 (80.89)	32 (7.94)	45 (11.17)
	A9* Disrespectful behavior among staff	256 (62.90)	70 (17.20)	81 (19.90)
Staffing and work pace	A2 Enough staff to handle workload	107 (25.85)	53 (12.80)	254 (61.35)
	A3* Staff work longer hours than best for care	67 (16.54)	66 (16.30)	272 (67.16)
	A5* Unit relies too much on temporary staff	153 (40.26)	99 (26.05)	128 (33.68)
	A11* Work pace too rushed affecting safety	195 (47.91)	59 (14.50)	153 (37.59)
Organizational learning–continuous improvement	A4 Unit reviews processes to improve safety	294 (71.53)	54 (13.14)	63 (15.33)
	A12 Safety improvement changes evaluated	280 (70.00)	73 (18.25)	47 (11.75)
	A14* Same patient safety problems continue	255 (64.89)	53 (13.49)	85 (21.63)
Response to error	A6* Mistakes held against staff	183 (46.68)	89 (22.70)	120 (30.61)
	A7* Reporting events feels like blaming staff	167 (43.72)	71 (18.59)	144 (37.70)
	A10 Unit focuses on learning rather than blame	241 (60.40)	60 (15.04)	98 (24.56)
	A13* Lack of support after safety errors	193 (48.01)	77 (19.15)	132 (32.84)
Supervisor/clinical leader support	B1 Supervisor considers staff safety suggestions	314 (77.53)	53 (13.09)	38 (9.38)
	B2* Supervisor encourages shortcuts when busy	178 (44.84)	77 (19.40)	142 (35.77)
	B3 Supervisor acts on safety concerns	332 (83.00)	27 (6.75)	41 (10.25)
Communication about error	C1 Staff informed about errors	298 (72.86)	70 (17.11)	41 (10.02)
	C2 Errors discussed to prevent recurrence	321 (79.26)	43 (10.62)	41 (10.12)
	C3 Staff informed about changes from reports	314 (77.53)	60 (14.81)	31 (7.65)
Communication openness	C4 Staff speak up about safety concerns	284 (70.12)	66 (16.30)	55 (13.58)
	C5 Staff speak up to authority about unsafe care	251 (65.19)	81 (21.04)	53 (13.77)
	C6 Leaders open to safety concerns	235 (62.17)	82 (21.69)	61 (16.14)
	C7* Staff afraid to ask questions	219 (55.58)	64 (16.24)	111 (28.17)
Reporting patient safety events	D1 Near-miss events reported	238 (62.96)	65 (17.20)	75 (19.84)
	D2 No-harm events reported	217 (59.62)	55 (15.11)	92 (25.27)
Hospital management support	F1 Management prioritizes patient safety	306 (74.45)	54 (13.14)	51 (12.41)
	F2 Management provides safety resources	221 (53.77)	75 (18.25)	115 (27.98)
	F3* Management interested only after adverse events	135 (33.75)	99 (24.75)	166 (41.50)
Handoffs and information exchange	F4* Information lost during patient transfer	214 (57.37)	78 (20.91)	81 (21.72)
	F5* Information lost during shift changes	237 (64.23)	54 (14.63)	78 (21.14)
	F6 Adequate time for information exchange	227 (61.02)	70 (18.82)	75 (20.16)

## References

[1] Kohn LT, Corrigan JM, Donaldson MS (2000). To Err is Human: Building a Safer Health System.

[2] Landrigan CP, Parry GJ, Bones CB, Hackbarth AD, Goldmann DA, Sharek PJ (2010). Temporal trends in rates of patient harm resulting from medical care. N Engl J Med.

[3] Jha AK, Larizgoitia I, Audera-Lopez C, Prasopa-Plaizier N, Waters H, Bates DW (2013). The global burden of unsafe medical care: analytic modelling of observational studies. BMJ Qual Saf.

[4] World Health Organization (2021). Global Patient Safety Action Plan 2021-2030: Towards Eliminating Avoidable Harm in Health Care. https://www.who.int/publications/i/item/9789240032705.

[5] Braithwaite J, Herkes J, Ludlow K, Testa L, Lamprell G (2017). Association between organisational and workplace cultures, and patient outcomes: systematic review. BMJ Open.

[6] Pronovost PJ, Berenholtz SM, Goeschel CA (2006). Creating high reliability in health care organizations. Health Serv Res.

[7] Sorra JS, Dyer N (2010). Multilevel psychometric properties of the AHRQ hospital survey on patient safety culture. BMC Health Serv Res.

[8] Waterson P, Griffiths P, Stride C, Murphy J, Hignett S (2010). Psychometric properties of the hospital survey on patient safety culture: findings from the UK. Qual Saf Health Care.

[9] (2026). Agency for Healthcare Research and Quality: Hospital survey on patient safety culture (SOPS hospital survey) version 2.0: user's guide. Agency for Healthcare Research and Quality (US), Rockville, MD. https://www.ahrq.gov/sops/surveys/hospital/index.html.

[10] Hedsköld M, Pukk-Härenstam K, Berg E, Lindh M, Soop M, Øvretveit J, Sachs MA (2013). Psychometric properties of the hospital survey on patient safety culture, HSOPSC, applied on a large Swedish health care sample. BMC Health Serv Res.

[11] Reis CT, Paiva SG, Sousa P (2018). The patient safety culture: a systematic review by characteristics of hospital survey on patient safety culture dimensions. Int J Qual Health Care.

[12] Elmontsri M, Banarsee R, Majeed A (2018). Improving patient safety in developing countries-moving towards an integrated approach. JRSM Open.

[13] El-Jardali F, Jaafar M, Dimassi H, Jamal D, Hamdan R (2010). The current state of patient safety culture in Lebanese hospitals: a study at baseline. Int J Qual Health Care.

[14] Bodur S, Filiz E (2010). Validity and reliability of Turkish version of "hospital survey on patient safety culture" and perception of patient safety in public hospitals in Turkey. BMC Health Serv Res.

[15] Camacho-Rodríguez DE, Carrasquilla-Baza DA, Dominguez-Cancino KA, Palmieri PA (2022). Patient safety culture in Latin American Hospitals: a systematic review with meta-analysis. Int J Environ Res Public Health.

[16] Nie Y, Mao X, Cui H, He S, Li J, Zhang M (2013). Hospital survey on patient safety culture in China. BMC Health Serv Res.

[17] Rajalatchumi A, Ravikumar TS, Muruganandham K, Thulasingam M, Selvaraj K, Reddy MM, Jayaraman B (2018). Perception of patient safety culture among health-care providers in a tertiary care hospital, South India. J Nat Sci Biol Med.

[18] Landefeld J, Sivaraman R, Arora NK (2015). Barriers to improving patient safety in India: focus groups with providers in the southern state of Kerala. Indian J Community Med.

[19] Krishnamoorthy Y, Govindan D, Rajaa S, Sinha I, Kanth K, Krishnan M, Samuel G (2023). Evaluation of National Patient Safety Implementation Framework in selected public healthcare facilities of Tamil Nadu: an operational research. J Patient Saf.

[20] Ammouri AA, Tailakh AK, Muliira JK, Geethakrishnan R, Al Kindi SN (2015). Patient safety culture among nurses. Int Nurs Rev.

[21] von Elm E, Altman DG, Egger M, Pocock SJ, Gøtzsche PC, Vandenbroucke JP (2007). The Strengthening the Reporting of Observational Studies in Epidemiology (STROBE) statement: guidelines for reporting observational studies. Lancet.

[22] Dean AG, Sullivan KM, Soe MM (2026). OpenEpi: Open Source Epidemiologic Statistics for Public Health, Version 3.01 (Internet). https://www.OpenEpi.com.

[23] Okuyama JH, Galvao TF, Silva MT (2018). Healthcare professional's perception of patient safety measured by the hospital survey on patient safety culture: a systematic review and meta-analysis. Sci World J.

[24] Weaver SJ, Lubomksi LH, Wilson RF, Pfoh ER, Martinez KA, Dy SM (2013). Promoting a culture of safety as a patient safety strategy: a systematic review. Ann Intern Med.

[25] Aiken LH, Clarke SP, Sloane DM, Sochalski J, Silber JH (2002). Hospital nurse staffing and patient mortality, nurse burnout, and job dissatisfaction. JAMA.

[26] Starmer AJ, Spector ND, Srivastava R (2014). Changes in medical errors after implementation of a handoff program. N Engl J Med.

[27] Morello RT, Lowthian JA, Barker AL, McGinnes R, Dunt D, Brand C (2013). Strategies for improving patient safety culture in hospitals: a systematic review. BMJ Qual Saf.

